# Association of Chromosomal Translocation and MiRNA Expression with The Pathogenesis of Multiple Myeloma

**Published:** 2014-05-25

**Authors:** Najmaldin Saki, Saeid Abroun, Saeideh Hajizamani, Fakher Rahim, Mohammad Shahjahani

**Affiliations:** 1 Research Center of Thalassemia and Hemoglobinopathy, Ahvaz Jundishapur University of Medical Sciences, Ahvaz, Iran; 2 Department of Hematology and Blood Banking, Faculty of Medical Sciences, Tarbiat Modares University, Tehran, Iran; 3 Toxicology Research Center, Ahvaz Jundishapur University of Medical Sciences, Ahvaz, Iran

**Keywords:** Multiple Myeloma, Prognosis, MicroRNAs (miRNA), Tumor Suppressor

## Abstract

Multiple myeloma (MM), is the second most common blood cancer after non-Hodgkin’s lymphoma. Genetic changes, structural and numerical chromosome anomalies, are involved in pathogenesis of MM, and are among the most important prognostic factors of disease-associated patient survival. MicroRNAs (miRNAs) are small 19-22 nucleotide single-stranded RNAs involved in important cellular processes. Cytogenetic changes in plasma cells alter miRNA expression and function. MiRNAs act as tumor suppressors and oncogenes by affecting intracellular signaling pathways. MiRNA expression is associated with a specific genetic change and may assist with diagnosis and disease prognosis. This study aims to evaluate recent findings in MM-associated cytogenetic changes and their relationship with changes in the expression of miRNAs. We have determined that MM-associated cytogenetic changes are related to changes in the expression of miRNAs and CD markers (cluster of differentiation) are associated with disease survival. Information about these changes can be used for therapeutic purposes and disease prognosis.

## Introduction

Multiple myeloma (MM), is a heterogeneous malignant neoplasm that occurs in patients over 50 years of age. This disease is the second most prevalent blood cancer (10%) after non-Hodgkin’s lymphoma (1). MM is a result of genetic mutations or changes, including chromosomal translocations, deletions and duplications in plasma cells. These genetic changes are structural (IgH translocation) and numerical variations [numerical aberration of single chromosomes or chromosomal regions (del 13q) or changes in ploidy] (2). Cytogenetic changes are among the most important prognostic factors in treatment of MM (3). MM has a variable prognosis where survival ranges from several months to more than ten years (4). Detection of genetic alterations is not only important for prognosis but can be used for both therapeutic purposes and monitoring prognosis. In addition, altered expression of microRNAs (miRNAs) in association with different cytogenetic and genetic abnormalities (5) can assist with diagnosis and disease prognosis. Cytogenetic changes in MM are associated with disease survival and changing patterns of miRNA and CD markers, in which these changes may be exploited for therapeutic and prognostic purposes (Table 1).

### Chromosomal translocation associated with good prognosis

#### Chromosomal translocation t(11;14)

The t(11;14) translocation is a structural genetic abnormality found in MM with a prevalence of 16% (6). This translocation leads to juxtaposition of cyclin D1 (CCND1) with the immunoglobulin heavy (IgH) chain loci and increasing plasma cell proliferative activity in the bone marrow (7). The t(11;14) translocation has a good prognosis and longer overall survival with better response to treatment (8). Recent studies have recognized significant increases in CCND1 expression as a favorable factor in this translocation (9). There is a link between the t(11;14) translocation and *P53* deletions, but not with retinoblastoma (*RB*) deletions (4). Bone disease is higher in the t(11;14) translocation and hyperdiploid multiple myeloma (H-MM) because of the lower invasion rate of monoclonal gammopathy of undetermined significance (MGUS) relative to MM with additional time for bone lesions to develop (10). This translocation is associated with CD117−, CD56− and CD20+ phenotype, in which the last two markers are associated with immature plasma cell morphology (11-13). CD56 [neural cell adhesion molecule (NCAM)] marker is expressed in 62-82% of plasma cells in MM patients and is involved in cell attachment to cellular components of the bone marrow microenvironment. Lack of CD56 expression is associated with aggressive signs with a high incidence in plasma cell leukemia (14-16). The phenotypic property of CD56− is suggestive of an involvement in disease progress, migration of myeloma cells out of the bone marrow, presence of Bence Jones protein, and may contribute to thrombocytopenia (17, 18). MGUS plasma cells do not express CD56 marker, hence this can be used to differentiate between them (19). The t(11;14) translocation is associated with lymphoplasmacytoid morphology and light chain lambda myeloma (20-22). High prevalence of this translocation has been reported in IgM, IgE and nonsecretory MM (23). There is a high prevalence of focal bone lesions in the patients who overexpress CCND1 (9). Increased expressions of miR-361-3p, miR-30e and miR-582-5p have been observed in the t(11;14) translocation. The first two miRNAs target the PPP2R4 gene, the activation of IL-6 signaling, and result in increased growth and survival of myeloma cells (24). Expression of CD23 (Fc receptor for IgE) has been suggested in association with an abnormality in chromosome 11, including the t(11;14) translocation (25). CD23 positive patients with t(11;14) translocation have thrombocytopenia with lower overall survival compared with CD23 negative patients (26). Although the role of CD23 marker in plasma cell leukemia with the t(11;14) translocation is not known, it may be considered a reasonable therapeutic for a novel target therapy (27).

**Table 1:** Cytogenetic abnormalities in MM


Abnormality	Upregulated oncogenes	CD marker	Ig isotype	Prognosis	References

**t(11;14)**	Cyclin D1	CD20+ CD56− CD117− CD23+	Light chain lambda Myeloma, IgM, IgE, non-secretory myeloma	Good	(7, 8, 11-13, 20-23, 25)
**t(4;14)**	MMSET FGFR3	CD221+ G-protein coupled receptor 5D	IgA isotype	Poor	(6, 22, 28-30)
**t(14;16)**	C-MAF WWOX Cyclin D2	CD221+	Free light chain, IgA myeloma	Poor	(1, 9, 20, 31-33)
**t(14;20)**	MAFB Cyclin D2	_	_	Poor	(9, 33, 34)
**Del 13p**	RB	CD33+ CD117−	Free light chain	Poor	(12, 20, 35-37)
**Del 17p**	TP53	_	_	Poor	(38, 39)
**1p del**	CDC14A	_	_	Poor	(40, 41)
**1q gain**	CKS1B	_	_	Poor	(40, 42)


Ig; Immunoglobulin, FGFR; Fibroblast growth factor receptor, MMSET; Multiple myeloma SET domain protein, MAFB; Masculoaponeurotic fibrosarcoma oncogene homologe B, WWOX; WW domain-containing oxidoreductase, RB; Retino blastoma and CKS1B; Cyclin kinase subunit 1B.

#### Hyperdiploidy

H-MM, the most common form of myeloma, is associated with trisomy in chromosomes 3, 5, 7, 9, 11, 15, 19, and 21, and less commonly with primary IgH translocations. H-MM is a numerical chromosomal abnormality associated with a favorable prognosis and better response to treatment (43, 44). There is high platelet count and bone involvement in H-MM patients, but the level of serum markers of bone diseases such as bone alkaline phosphatase (BAP), carboxy-terminal telopeptide of type I collagen (ICTP), osteocalcin (OC), carboxy-terminal propeptide of type I collagen (PICP) and tartrateresistant acid phosphatase (TRAP) is similar to non-H-MM patients. Chromosome 13 deletion is not effective on overall survival (OS) and progression- free survival (PFS) of H-MM patients (10). Decreases in miRNA-24, -152 and -425 increase the expression of CCND1, fibroblast growth factor receptor 3 (*FGFR3*), transforming acidic coiledcoil- containing protein 3 (TACC3) and masculoaponeurotic fibrosarcoma oncogene homolog B (MAFB) genes in H-MM (45).

#### Chromosomal translocation t(6;14)

The t(6;14) translocation is present in 3% of MM patients, involves the cyclin D3 pathway in myeloma, and is associated with a good prognosis after treatment (1, 34, 46). This translocation may lead to juxtaposition of multiple myeloma oncogene 1/ interferon regulatory factor 4 (MUM1/IRF4) with the IgH loci and deregulation of its expression (47). This translocation has also been reported in plasma cell leukemia and non-Hodgkin’s lymphoma (48).

### Chromosomal translocation associated with poor prognosis

#### Chromosomal translocation t(4;14)

The t(4;14) translocation observed in 13-15% of MM patients is associated with short survival and poor response to chemotherapy (23, 49, 50). Chromosome 13 deletion is highly prevalent in this translocation. This translocation can affect *FGFR3* and multiple myeloma set domain (*MMSET*), Wolf-Hirschhorn syndrome candidate 1 (WHSC1) genes, which have been introduced as MM-associated oncogenes, and have been involved in MGUS conversion to MM (28, 29, 51). Increased expression of *FGFR3* and *MMSET* has been observed in 70 and 100% of MM patients with the t(4;14) translocation, respectively. *MMSET* is a surrogate marker for this translocation, and can be used for its detection (2, 52). No differences in survival rates between MM patients with and without increased *FGFR3* level have been observed (53). This translocation is associated with the IgA isotype, increased expression of G-protein coupled receptor 5D, and an immature morphology. Short survival and poor prognosis are associated with IgA isotype and G-protein coupled receptor 5D, respectively (6, 22, 28, 30, 54). A strong relationship has been found between the t(4;14) translocation and *RB* deletions (79%) in the Gutiérrez et al. (4) study however the final result showed that the presence of this translocation alone was a poor prognostic factor and decreased survival and simultaneous presence of *RB* deletions were not effective on prognosis. Expression of an insulin-like growth factor (IGF) receptor such as CD221 on MM cells has been associated with poor prognosis and short survival, which its high levels have been observed with 14q32 translocations including t(4;14) and t(14;16) (31). IGF-1 is considered a factor for MM cell growth and survival due to increases in expression of the CD221 marker. This factor increases the activity of interleukin-6 (IL-6), a growth and survival factor in MM, and plays an important role in angiogenesis by stimulating secretion of vascular endothelial growth factor (VEGF) through the MEK/ ERK pathway in myeloma cells (52, 55-58). MiR- 126 has been recognized as a regulator of *MMSET*. *MMSET* increases the proliferation of MM cells by stimulating the expression of myelocytomatosis oncogene (c-MYC) (59). Decreased expression of MiR- 146a and MiR-135b in t(4;14) translocation leads to increases in expression of *PELI2* and IRAK1 (IL-1 receptor associated kinase) genes, which are involved in the IL-1 signaling pathway, with an eventual increase in expression of IL-6 and MM cell growth (60). High levels of miR-99b and miR-125a-5p are also related to this translocation. miR-125a-5p deregulates the expression of BAK1 (Bcl-1 homologous antagonist/ killer), Kruppel-like factor 13 (KLF13) and ERBB2/3 (epidermal growth factor receptor; EGFR) genes (24, 61, 62).

#### Cytogenetic abnormalities of chromosome 13

This disorder mainly involves monosomy in chromosome 13, however 13 deletion and 13q translocations are present in less than 15% of MM patients. The characteristic feature of this abnormality is short event-free survival (EFS), short OS, rapid recurrence and resultant unfavorable prognosis (63, 64). Chromosome 13 deletion or monosomy 13 is an adverse prognostic factor that occurs in 30-50% of MM patients (35). Monosomy 13 plays a role in MGUS conversion to MM in patients who have a previous MGUS history, and usually occurs before IgH translocation (47, 65). Previously, a respective 82 and 100% significant association between t(4;14) and t(14;16) translocations with chromosome 13 abnormalities was observed (6). Although the chromosome 13 deletion appeared to be associated with t(4;14), t(14;16), t(14;20) translocations and del (17), it was not recognized as an independent prognostic factor (1, 66, 67). Ploidy status had no effect on EFS and OS rates in MM patients with this chromosomal deletion and a 13q abnormality would not alter OS in hyperdiploid and hypodiploid patients (67-69). The presence of del (13q14) has been shown to be a factor of poor response to treatment; myeloma cells with this deletion have higher proliferative capacity compared with cells without it (70). Perhaps the presence of the *RB* gene on 13q chromosome has caused a reduction in the *RB* level following del13q and increased expression of IL-6 in myeloma cells, which would result in higher invasiveness of the disease (47, 36). However, this was not observed in a study by Zojer et al. (70). *RB* deletions are associated with bone disease, low hemoglobin (Hb) levels, and has no effect on the outcome of t(11;14), t(4;14) and t(14;16) translocations (4). Higher free light chain levels have been observed in MM patients with chromosome 13 deletion compared with patients without such abnormalities (20). Chromosome 13 deletion (13q) has a higher incidence in CD33− positive and CD117- negative patients associated with poor prognosis (12, 37). CD117 marker (C-kit) is a transmembrane glycoprotein member of the subclass III receptor tyrosine kinase family. CD33 (myeloid cell marker) is a 67 kDa glycoprotein member of the sialo-adhesion molecule family, which is not expressed in normal plasma cells. Serum levels of β2-microglobulin and lactate dehydrogenase are higher in CD33− positive patients (71, 72). The miR-16-1 and miR-15a genes are located on chromosome 13 and are not expressed in a chromosome 13 deletion. Increased expression of these miRNAs will induce apoptosis, thus they can be presumed to be tumor suppressors (5, 73). Expressions of miR-18, miR-19 and miR-20 in chromosome 13 deletions are higher than in patients without the deletion. Expression of miR-15a, miR-16-1 and miR-17-92 is independent of chromosome 13 deletion, and has been suggested to be associated with poor prognosis (5, 74, 75). Chromosome 13 deletion has been reported to be associated with decreased expression of miR-221 (61).

#### Del 17p13

This deletion occurs in 11% of MM patients. It inactivates *P53* and has a poor prognosis for treatment. There is a strong correlation between this deletion and t(14;16) but not with t(4;14) translocation (2, 32, 66). Lodé et al. (76) have shown that 37% of MM patients with del 17p had TP53 mutations, while none of the patients without this deletion had this mutation, which supported the association of del 17p with TP53 mutations. Xiong et al. (38) have reported decreased expression of TP53, an adverse prognostic factor, which can be considered as a surrogate for detection of del17p. However Chng et al. (28) found no significant relationship for survival in MM patients with reduced TP53 level compared to those without it. This was not an independent prognostic factor (28, 69), hence there is a requirement for further studies in this area. There is a relationship between TP53 deletions and expression of *P53* nuclear protein with poor prognosis (77). Chang et al. (39) have reported that all MM patients with nuclear expression of *P53* were also positive for TP53 deletion; therefore, the nuclear expression of *P53* could be helpful to predict del17. Chen et al. (78) stated that nuclear expression of *P53* has been considered as a surrogate for del17p in relapsed/refractory patients who receive lenalidomide plus dexamethasone. Moreover, aberrant expression of *P53* also plays a role in the development of extramedullary MM (17). There is a similar incidence of del17p in H-MM and non-H-MM (10). Although no clinical and laboratory relationship with *P53* deletions have been found, del17p13 is characteristically related with hypercalcemia, soft-tissue plasmacytomas and a high level of sIL6-R (4, 32). Decreased expression of mir-192, mir-194 and mir-215 in MM with increasing MDM2 (a Tp53 inhibitor) results in increased oncogenic potential in myeloma cells, and these miRNAs are presumed to have a tumor suppressor effect (79).

#### Chromosome 1 abnormality

Chromosome 1 abnormality occurs in the form of 1p deletion and 1q amplification (gain of 1q) in 18 and 38% of MM patients with poor prognosis, respectively (40). A gain of 1q increases the expression of cyclin kinase subunit 1B (*CKS1B*) which is associated with bone marrow plasmacytosis, pathogenesis and disease progression as well as del 13 and del *P53* (42, 80). *CKS1B* amplification along with high plasma cell labeling index (PCLI) is a marker of MM proliferation and short survival; thus far, there is a reported association with t(4;14), del13 and *MAF* translocation, but no association with t(11;14) (35, 81). 1p21 deletion involves *CDC14A* deletion with a poor prognosis. The level of C-reactive protein (CRP) is high in these patients and has been reported to be associated with t(4,14), del 17p and del 13q (40, 41). In addition to *CDC14A* deletion, deletions of *CDKN2C, FAF1, MTF2, TMED5, FAM46C* and *VSP33* genes on 1p have been observed and are associated with a poor prognosis. *CDKN2C* homozygous deletion increases proliferation and results in poor prognosis (79, 82).

#### t(14;16)/t(14;20) translocation

The t(14;16) translocation occurs in 5% of MM patients and is associated with poor prognosis and reduced OS, leading to deregulation of the C-maf proto-oncogene (1). The c-MYC is a basic zipper transcription factor involved in numerous cellular processes such as proliferation, differentiation and production of IL-6. Increased expression of this factor results in increasing growth and survival of myeloma cells, their attachment to bone marrow stromal cells, tumor formation and VEGF secretion through targeting CCND1, integrin *β7*, and *CCR1* (chemokine receptor) genes. Recently, disruption of the WW domain-containing oxidoreductase (*WWOX*) gene has been reported. This gene is located on chromosome 16, and has recently been recognized as a new tumor suppressor (1, 32, 83-86). The t(14;16) translocation has a higher prevalence in IgA myeloma and the level of free light chain is high in IgH translocations, especially in t(14;16) (20, 33). Upregulation of miR-1 and miR-133a in connection with the t(14;16) translocation has been reported; miR-1 gene deregulates expression of *TAGLN2, KLF4* and *c-MET* genes (62). The t(14;20) translocation is associated with poor prognosis and occurs in 0.9-1.5% of MM cases, in which there is increased expression of the MAFB oncogene (33, 34). MAFB is a member of the basic/leucin zipper transcription factors and a proto-oncogene product. Suzuki et al. (87) have found that increased expression of MAFB and other members of the above mentioned family in myeloma cells increases the expression of ARK5 [AMP-activated protein kinase (AMPK)-related protein kinase] leading to increased invasion of myeloma cells through the IGF-1/AKT induced cell invasion pathway. Expression of cyclin D2 (CCND2) is deregulated in patients who have the t(14;16) and t(14;20) translocations (9). MiR-133b expression is also increased in these translocations (24).

#### Deregulation of miRNA expression in multiple myeloma

**Fig 1:**
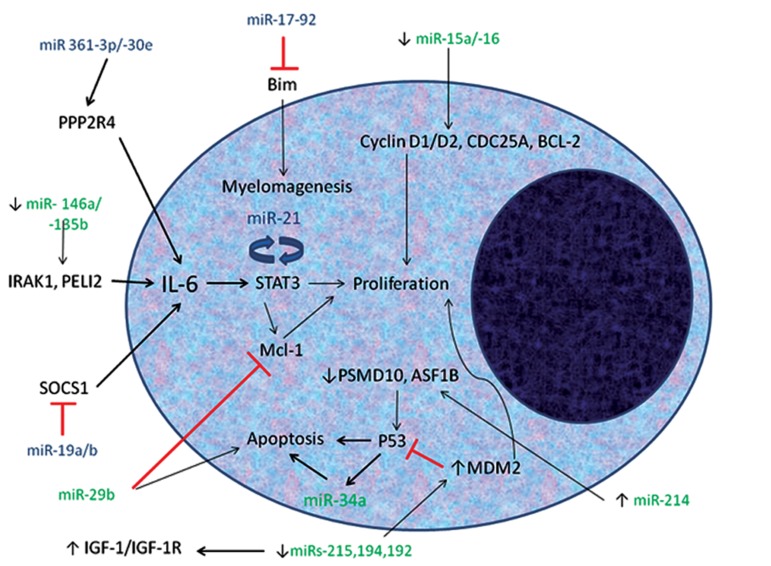
MicroRNAs (miRNAs) are involved in pathogenesis of multiple myeloma (MM) by targeting some signaling pathways in myeloma cells. Oncogenic miRNAs are shown in blue and tumor suppressive miRNAs are shown in green. Expression of 361-3p/-30e, 19a/b and 17-92 miRNAs as oncogenes are increased in myeloma cells. The first two miRNAs respectively target the PPP2R4 and SOCS1 genes and activate the IL-6 signaling pathway, eventually augmenting the growth of myeloma cells. Expression of 17-92 miRNA in myeloma cells prevents their apoptosis by reduced expression of Bim. -15a/-16, -215-194-192, -146a/-135b,-34a,-29b and -214 miRNAs have been identified as tumor suppressors in MM. Decreased level of -215-194-192, -146a and -15a/-16 miRNA results in increased expression of MDM2, IRAK1/PELI2 and cyclin D1/D2, respectively, with increased growth of myeloma cells as a result. Increased miRNA-214 can induce the expression of p53 and apoptosis of myeloma cells by reducing the expression of PSMD10 and ASF1B. Expression of miRNA-29b and miRNA-34a are decreased in myeloma cells; miRNA-29b causes apoptosis by reducing expression of Mcl-1 involved in the IL-6 pathway. IRAK1;IL-1 receptor associated kinase, PELI2; Protein pellino homolog 2, SOCS1; Suppressor of cytokine signaling 1, IGF- 1/IGF-1R; Insulin-like growth factor/receptor, MCL-1; Myeloid-cell-leukemia, MDM2; Mouse double minute 2 homolog, CDC25A; Cell division cycle 25 homolog A, BCL-2; B-cell lymphoma 2, PPP2R4; Protein phosphatase 2 activator regulatory subunit 4 and ASF1B; Anti-silencing function 1 homolog B.

MiRNAs are small, 19-22 nucleotide RNA molecules, which regulate processes such as development, proliferation, differentiation and apoptosis in cells (88). Due to genetic changes in MM, miRNA expression and function is altered. This alteration has an important role in pathogenesis of MM and may function as a tumor suppressor or oncogene (Table 2) (89). Therefore, measurement of miRNA expression may help in determining disease prognosis (Fig 1). Increased expression of miR-19a/b in MM cases has indicated the role of these RNA molecules in activation of JAK/STAT signaling and in expression of IL-6 during negative regulation of SOCS1, which has been shown to be important in MM pathogenesis as an oncogene (90). Mir-21 is also controlled in MM through the STAT pathway and has a role as an oncogene (90, 91). IL-6 causes the growth of myeloma cells in the JAK/STAT pathway by increasing expression of myeloid-cell-leukemia (Mcl-1). MiR-29b, as a tumor suppressor, inhibits this pathway by decreasing Mcl-1 and causes apoptosis by increasing caspase 3 activity which is reduced in MM (61, 92). MiR-15a/16 has anti-proliferative and tumor suppressive roles by inhibiting CDC25A, cyclin D1/D2 and Bcl2. Decreased expression of MiR-15a/16 in MM contributes to proliferation and survival of MM cells with increasing activity of the Notch, c-jun and TP53 pathways (5, 73, 93). Increased expression of miR-17-92 is involved in anti-apoptotic signaling by decreasing Bim (90). The expression of this miRNA is significantly increased in MM but not MGUS, indicating its possible role in transformation of MGUS to MM and its suitability as a marker for the diagnosis and treatment of MM patients (88, 94, 95). Re-expression of miR-214 induces the expression of *P53*, increases CDKN1A and results in eventual apoptosis of MM cells by downregulating PSMD10 (which encodes the oncoprotein gankyrin) and ASF1B (a histone chaperone required for DNA replication). Therefore, this miRNA also has a tumor suppressor role (96). Mir-124-1 downregulates CDK6 and decreases phosphorylation of RB and acts as a tumor suppressor. Despite methylation of this miRNA in MM cell lines, the methylation has not been observed in bone marrow samples, which indicates the lack of an important role for it in pathogenesis and progression of MM (97). miR- 148a/181a/20a/221/625/99b overexpression has been observed in MM patients, among which miR20a and miR-148a are associated with decreased survival (61). *P53* induces the expression of miR-192, miR-194 and miR-215 which are decreased in MM patients. Their re-expression in MM cells decreases MDM2 and increases *P53* which inhibits cell growth. Hence they can be considered tumor suppressors (98). MiR-34a is the *P53* target that causes apoptosis, indicating its tumor suppressor role. This miRNA is not methylated in normal blood cells, but it is hypermethylated in MM cell lines (99). Decreased expression of miR-30b in cultured plasma cells has caused its identification as a tumor suppressor (91). Decreased expression of miRNA- 196b, 135b, -320, -20a, -19b,-19a,-15a increase CCND2 expression. CCND2 is involved in regulation of progression from G1 to the S phase of the cell cycle, and has been known as a transcriptional target for *MAF* protein (9, 60).

**Table 2:** Deregulated microRNA expression in multiple myeloma and their biological functions


MicroRNA	Biological function in MM	Cytogenetic abnormality	References

**miR-361-3p miR-30e miR-582-5p**	Oncogene	t(11;14)	(24)
**miR-126**	Oncogene	t(4;14)	(49)
**miR-99b miR-125a-5p**	Oncogene	t(4;14)	(24),(51)
**miR-mir133a/b**	Oncogene	t(14;16),t(14;20)	(52)
**miR-1**	Oncogene	t(14;16)	(52)
**miR-18/-19/-20**	Oncogene	Del 13p	(5),(67),(68)
**miR-19a/b**	Oncogene	_	(90)
**miR-21**	Oncogene	_	(90),(91),
**miR-17-92**	Oncogene	_	(90)
**miR-425/-152/-24**	Tumor suppressor	HRD	(30)
**miR-146a/-135b**	Tumor suppressor	t(4;14)	(50)
**miR-215/-192/-194**	Tumor suppressor	Del 17p	(75),(98)
**miR-15a/16**	Tumor suppressor	Del 13p	(5),(66)
**miR-221**	Tumor suppressor	Del 13p	(51)
**miR-29b**	Tumor suppressor	_	(51),(92)
**miR-214**	Tumor suppressor	_	(96)
**miR-124-1**	Tumor suppressor	_	(97)
**miR-34a**	Tumor suppressor	_	(99)
**miR-30b**	Tumor suppressor	_	(91)


MM; Multiple myeloma, miR; microRNA, Del; Deletion and HRD; Hyperdiploidy.

## Discussion

Thus far, MM is an incurable disease. Patients who undergo high dose chemotherapy or transplantation survive for five years (100). MMassociated genetic changes can be used not only to determine the disease prognosis, but a proper therapeutic approach based on mutation and cytogenetic changes to increase patient survival. This article has reviewed these genetic changes as well as altered miRNA expression in disease prognosis. For example, t(11;14), t(6;4) translocations and H-MM have good prognosis whereas t(4;14), del13, del17, and chromosome 1 abnormality show shorter patient survival and poor prognosis. Hypodiploid myeloma and 11q abnormalities are other genetic variations associated with poor prognosis in MM (101-103). Chang et al. (104) have reported a higher incidence of these genetic changes in plasma cell leukemia compared to MM, where this condition may assist in determining disease prognosis and treatment. Chiecchio et al. (105) have also confirmed a higher incidence of the t(11;14), t(14;16) translocations and del16q in plasma cell leukemia (PCL), which may be involved in different clinical symptoms of PCL and MM. Genetic changes in MM result in altered expression and function of miRNAs. These changes are also present in other blood diseases, including Waldenstrom’s macroglobulinemia (WM), acute myeloid leukemia (AML) and chronic lymphocytic leukemia (CLL). For example, low expression of miR-15a/16 in AML and CLL is associated with disease prognosis as well as patient survival (106). Increased miR-363/-206/-155 and decreased miR- 9 are important prognostic markers in WM (107), but their role in MM is unclear and need further investigations. CD marker expression differs between plasma cells and MM cells. For example, CD200 is not normally expressed on plasma cells, but its expression is associated with poor prognosis and short post-treatment EFS. If the CD200 marker is not expressed, the patient will have better EFS (108, 109). CD45 negative is a progressive phenotype of MM. MM patients who have this phenotype show characteristically shorter overall survival compared to the CD45 positive phenotype (110). The effect of IGF-1 is dependent upon the expression of CD45 in MM cells, in which CD45 phosphatase inhibits the growth of MM cells by inhibiting IGF-1 signaling. Therefore, lack of CD45 may lead to a higher capacity for cell growth and survival (57).

## Conclusion

Genetic alterations of plasma cells in MM change the expression of such molecules as cyclins, miRNAs and CD markers relative to normal status, eventually augmenting the growth and survival of myeloma cells in the bone marrow. Sufficient information about the alterations in expression of the mentioned molecules and determining their relationship with genetic changes in MM can contribute to diagnosis, prognosis, pathogenesis and even treatment of this disease, for which further studies are required.
